# Physical activity, screen time, dietary habits, and health outcomes among children and adolescents in the Middle East and North Africa region: a narrative review

**DOI:** 10.3389/fpubh.2025.1628904

**Published:** 2025-09-24

**Authors:** Shaikha Eisa Alnaqbi, Rahab Sohail, Hadia M. Radwan, Maysm N. Mohamad, Falak Zeb, Haydar Hasan, Mona Hashim, Tareq Osaili, Sharifa AlBlooshi, Ayesha S. Al Dhaheri, Lily Stojanovska, Leila Cheikh Ismail

**Affiliations:** ^1^Department of Clinical Nutrition and Dietetics, College of Health Sciences, University of Sharjah, Sharjah, United Arab Emirates; ^2^Department of Nutrition, Al Qassimi Women's and Children's Hospital, Emirates Health Services (EHS), Sharjah, United Arab Emirates; ^3^Department of Nutrition and Health, College of Medicine and Health Sciences, United Arab Emirates University, Al Ain, United Arab Emirates; ^4^Research Institute for Medical and Health Sciences, University of Sharjah, Sharjah, United Arab Emirates; ^5^Department of Health Sciences, College of Natural and Health Sciences, Zayed University, Dubai, United Arab Emirates; ^6^Institute for Health and Sport, Victoria University, Melbourne, VIC, Australia; ^7^Nuffield Department of Women's & Reproductive Health, University of Oxford, Oxford, United Kingdom

**Keywords:** children, adolescent, physical activity, screen time, Arab region

## Abstract

**Background:**

Physical activity (PA) and screen time (ST) are crucial determinants of health among children and adolescents. The Middle East and North Africa (MENA) region has witnessed rapid urbanization, lifestyle transitions, and increased digitalization; all impact PA and ST behaviors. To our knowledge, we aimed to explore the prevalence of PA and ST among children and adolescents in the MENA region, as well as their correlations with health outcomes and dietary practices.

**Methods:**

A systematic search was conducted in major databases (Google Scholar, MEDLINE, EMBASE, SPORTdiscus, CINAHL, PsycINFO, and Scopus), 18 eligible studies from 7 countries were included. This narrative review explores the current literature on PA and ST patterns among children and adolescents in MENA, highlighting key trends, determinants, and health outcomes (dietary habits, obesity and overweight, body satisfaction, and quality of life).

**Results:**

Studies indicate that a significant proportion of children and adolescents in the region fail to meet the recommended PA guidelines, with sedentary lifestyles becoming increasingly prevalent. High ST exposure, driven by social media, gaming, and academic screen use, has been linked to unhealthy dietary habits, obesity, and metabolic disorders. This review highlights that boys are generally more physically active than girls. Socioeconomic factors, cultural norms, environmental barriers, and educational demands play crucial roles in shaping PA and ST behaviors.

**Conclusions:**

School-based interventions, parental influence, and policy measures promoting active lifestyles and accountable screen use are essential to mitigating the negative health effects. However, research gaps persist, particularly in longitudinal studies and intervention effectiveness. Addressing these challenges requires a multidisciplinary approach involving policymakers, educators, health professionals, and communities to foster healthier lifestyles among children and adolescents in the MENA region.

## 1 Introduction

Sedentary lifestyle practices including low physical activity (PA) and prolonged screen time (ST) are considered the major public health problems in the pediatric population of developing and developed countries (1). In recent decades, urbanization, technological, and transportation advancements have resulted in increased levels of sedentary behavior in the region. Shifts in work environments (working from home, extensive use of telecommunication, etc.) have increased the amount of time people spend sitting and reduced daily energy expenditure has been linked to increased body weight over time (2). In 2022, there were an estimated 390 million overweight children and adolescents in the world according to the World Health Organization. The percentage of children and adolescents who are overweight and/or obese has increased significantly from 8% in 1990 to 20% in 2022 (3). Diabetes and obesity rates in the countries of the MENA region are among the highest in the world (4). The 2021 Kids Nutrition and Health Survey (KNHS) in the United Arab Emirates (UAE) assessed 690 children aged 4 to 12.9 years, revealing that 28% were overweight or obese (5).

Worldwide, the majority of teenagers do not engage in physical activity, even though doing so has numerous health benefits. Approximately 84.7% of girls and 77.6% of boys between the ages of 11 and 17 do not engage in regular physical activity (6). Global statistics revealed that a considerable percentage of children and adolescents surpass the prescribed screen time recommendations. A multinational study involving 11,434 children aged 4–17 years across multiple nations, revealed that at least two-thirds of participants exceeded 2 h of daily screen time. Significantly, boys, overweight or obese children, and those with less parental education were more prone to surpass this barrier (7). Recent data from the WHO Regional Office for Europe, gathered in 2022, indicates a significant increase in problematic social media usage among adolescents, rising from 7% in 2018 to 11% in 2022. Furthermore, 34% of adolescents indicated daily participation in digital gaming, with more than 22% playing for a minimum of 4 h on gaming days (8). In the MENA region, the prevalence of physical inactivity and prolong screen time among children and adolescent is also an alarming public health issue. In Qatar, the proportion of students who spent more than 2 h on their screens ranged from 43% to 57% during the week and 50% to 62.5% on weekends (9). The prevalence of Iranian adolescents aged 13–18 years engaging in television viewing for two or more hours was 57.22% for girls and 57.57% for boys; personal computer usage for two or more hours was 10.31% for girls and 18.07% for boys; and low physical activity was reported at 39.34% for girls and 34.5% for boys (10). Most Arab adolescents do not meet the daily physical activity requirements. It has been reported that more than 85% of girls and 75% of boys aged 13–15 years in seven Arab countries (Djibouti, Egypt, Jordan, Libya, Morocco, Oman, and the United Arab Emirates) did not involve in the recommended amount of physical activity (at least 60 min per day) (11).

A sedentary lifestyle is one of the health behavior risk factors that have been attributed to abnormal health outcomes (12). Physical activity and exercise are crucial components of weight management programs for children and adolescents with overweight and obesity, providing numerous health benefits: enhanced physical fitness, cardiometabolic health, bone health, cognitive results, mental health, and decreased adiposity (13). In a cross-sectional study conducted in an urban region, researchers discovered that a general lack of physical activity was a predictor of obesity in adolescents (14). For adolescents, insufficient physical activity is defined as engaging in less than 150 min of moderate-intensity activity per week. Therefore, sedentary behavior has associations with general and cardiovascular disease mortality, diabetes, and obesity (15).

Screen time (ST), which includes watching television and playing electronic games, is seen as an alternative indication of inactivity. The American Academy of Pediatrics advises against screen time for children under the age of two and limits screen time for all children (16). Children spend the majority of their waking hours (50%−80%) engaged in inactive behaviors (17). Prolonged screen time is a marker of sedentary behavior and implies situations with low energy expenditure and lack of activity and is considered unhealthy behaviors (12). Sedentary habits, particularly prolonged screen time (ST), leisure time spent watching TV, and working on a computer, are indicated as risk factors for NCDs, which have their origins in early life (18). Excessive screen-based sedentary behavior, coupled with inadequate physical activity, is linked to a wide array of physical and psychological illnesses that can adversely impact health and wellbeing (19).

The most often mentioned obstacles to physical exercise were a lack of time, an inadequate sports facility, a lack of social support and motivation, gender and cultural standards, severe weather, and an oppressively hot temperature. An individual's level of physical activity is positively correlated with their motivation to lose or maintain weight, their gender, their eating habits, their leisure time activities, and their Body Mass Index (2). Some studies report contradictory findings, suggesting that screen time and physical activity may influence health outcomes independently and should be considered as two distinct entities with independent measurements (12). Health-related quality of life (HRQoL) is considered a multifaceted concept of physical, psychological, and social aspects of health that include experiences, beliefs, and perceptions (20). Sedentary life and prolonged screen time have been proposed as predictors of HRQoL in children and adolescents. Both are implied as unhealthy behaviors with their longitudinal trends that could persist into adulthood. It has also been the subject of research exploring its effects on the mental and psychological wellbeing of adolescents (21).

Furthermore, low PA participation is concerning because it may have a negative impact on children's and youth's mental health and quality of life. While some MENA countries are increasing the number of parks and improving access to sports facilities for their citizens, others are falling behind. To be successful in adopting interventions that promote PA, countries in the MENA region must first understand the obstacles and facilitators of PA programs within their respective populations (2). Therefore, we conducted this review to explore the physical activity level and screen time among children and adolescents in the MENA region.

## 2 Methods

This narrative review was conducted in accordance with the Preferred Reporting Items for Systematic Reviews and Meta-Analyses (PRISMA) guidelines (22).

### 2.1 Eligibility criteria

Studies having eligible participants included healthy children (5–12 years) and adolescents (12–18 years). Overweight and/or obese children and adolescents were also included. To be included, studies had to be peer-reviewed, published, written in Arabic or English and reported subjective or objective measurement of PA or ST or their combination. Gray literature, student dissertations or conference abstracts were excluded. The main outcomes were quality of life, obesity/overweight, life satisfaction, self-rated health or health self-perception, and food habits and behaviors. The review was limited to full manuscripts. There was no minimum sample size. All study designs were included.

### 2.2 Search strategy

**Figure 1:**
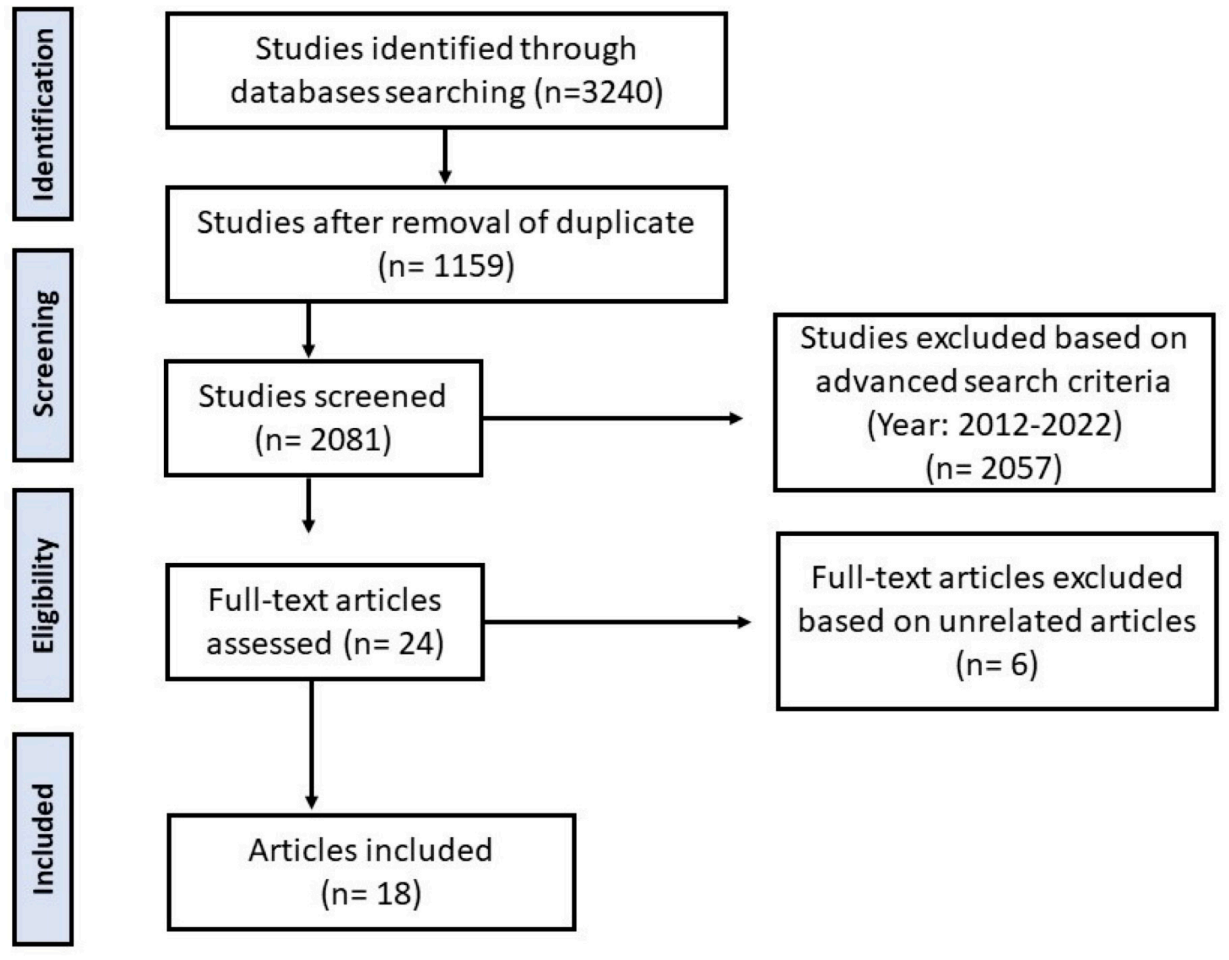
Flowchart of the study selection process. From 3240 studies identified, 1159 remained after duplicate removal. After screening 2081 studies, 2057 were excluded based on criteria from 2012-2022. Twenty-four full-text articles were assessed, with six excluded as unrelated. Eighteen articles were included in the final analysis. Flow chart of literature search.

We searched using databases Google Scholar, MEDLINE, EMBASE, SPORTdiscus, CINAHL, PsycINFO, and Scopus in March 2023, to identify relevant articles. A total of 3,240 articles were retrieved with the key terms including “physical activity,” “exercise,” “sport,” “screen time,” “sedentary,” “watching TV,” “kids,” “adolescent,” “children,” “MENA,” “EMRO,” “Arab,” and “gulf.” After customizing the year range between 2012 and 2022, a total of 2057 articles were excluded. Upon advanced search, we excluded unrelated articles (*n* = 6) about diseases, sleep, and socioeconomic correlates. Hence, a total of 18 related articles that met our inclusion criteria were used in this review (Figure 1).

### 2.3 Data extraction

Studies were imported into Endnote X9 software (Thomson Reuters, San Francisco, CA, USA). After de-duplication, four authors (S.A, R.S, LCI, and F.Z) screened titles and abstracts for relevant studies. Full-text copies of the eligible studies were assessed for final inclusion. Any disagreement between the four authors was resolved through a discussion and, when necessary, included a fourth author. The reference lists of all included studies were screened for additional studies not listed in the database search. Data were extracted for each study using an Excel spreadsheet. The extracted information included article, author, study design, publication year, location, sample size, age, mean age, gender, outcomes and measures, study instrument, and results (Table 1). Due to the variability in definitions, cut-off points, and measuring instruments employed in the studies, recalculation of aggregated prevalence estimates was not performed. The results were synthesized narratively instead.

**Table 1:** Characteristics and findings of the included studies.

**Reference/ country**	**Study design**	**Sample size (%/n girls) mean age/age range (years)**	**Type of behavior**	**Exposure and assessment instrument**	**Outcomes**	**Statistical analysis**	**Main results**
Motamed-Gorji et al. (21)/Iran	Cross-sectional	23,043 students (Girls: 49.2%) aged 6–18 years	ST and PA	- World Health Organization Global School-based Student Health Survey (GSHS) questionnaire - Physical Activity Questionnaire for Adolescents (PAQ-A) - Pediatric Quality of Life Inventory (PedsQL™ 4.0) - Physical activity questionnaire for children (PAQ-C) [low PA level (PAQ-A score: 1–1.9), and high PA level (PAQ-A score: 2–5)] [high ST (≥ 2 h per day) and low ST (< 2 h per day)]	Quality of life/Health-related quality of life (QoL/HRQoL)	Linear regression analysis	- Boys were substantially more physically active than girls (86.2%); 40.9% of the whole group had excessive screen time (ST 2 h) - ST duration had a significant inverse association with total QoL (β = – 0.49, *p* < 0.05). PA showed positive significant associations with HRQoL total score (β = 1.8, *p* < 0.05)
Matin et al. (12)/Iran	Cross-sectional	14,486 students, (49.23) 12.47/−18 years	PA and ST	WHO Global School-Based Student Health Survey (WHO-GSHS)	Life satisfaction and self-rated health	Multivariate analysis	−21.9% of boys reported more than 2 h of screen time compared to 15.2% of girls and 71.2% of boys and 60.4% of girls reported moderate to high physical activity - High physical activity was associated with good self-rated health (OR 1.37) and life satisfaction (OR 1.39), while prolonged screen time was not associated with good self-rated health (OR 1.02) and life satisfaction (OR 0.94)
Moradi et al. (17)/Iran	Cross-sectional	2,506 students, aged 10–12 years	PA and ST	International Modifiable Activity Questionnaire for Adolescents (MAQ) and Children	Overweight/Obesity	Logistic regression	−47.28% (95% CI: 45.33–49.24) of the participants spent more than 2 h a day on television and video watching and electronic games playing. - Participants with sufficient activity were 59.09%, while those with insufficient activity were 40.90% - People who spend greater time on ST activities, independent of their physical activities, are more susceptible to overweight and obesity *(p* = 0.002)
Alfaleh et al. (44)/Kuwait	Case-control	79 students, 6–10 years	ST and PA	- Eight-week nutrition and physical activity intervention - Modified healthy habit survey	Healthy eating habits and nutrition knowledge	Independent *t*-test McNemar's test	- Physical activity increased and screen time decreased in the intervention group. - A significant rise in nutrition knowledge scores (from 4.31.7 to 10.51.2) while Healthy eating improved considerably (from 12.81.8 to 14.51.5)
Allafi et al. (23)/Kuwait	Cross-sectional	906 adolescent students (443 girls), aged 14–19 years	PA	- A validated self-report questionnaire (ATLS) - American Academy of Pediatrics guidelines of a maximum of 2 h/d	Eating habits	Generalized linear mixed-effect models	−44·6% of the boys and 76·0% of the girls did not meet the recommended daily physical activity levels (≥2,520 MET-min/week, moderate to vigorous intensity). - 96·3% of boys and 96·7% of girls reported spending >2 h/d on screen time, with girls found to spend more time per day watching television (*P* = 0·02) and using a computer (*P* < 0·001). - The majority of the adolescents reported skipping breakfast and not having milk and milk products, vegetables and fruit daily, while nearly two-thirds of the boys and girls had sugar-sweetened drinks for more than 3 days/week
Al-Haifi et al. (31)/Kuwait	Cross-sectional	906 adolescent students (443 girls), aged 14–19 years	PA and ST	- A validated self-report questionnaire (ATLS) - The International Obesity Task Force (IOTF) age- and sex-specific BMI cutoff reference standards - American Academy of Pediatrics guidelines of a maximum of 2 h/d	Obesity	General linear model (GLM)	- Among boys, moderate and vigorous activities were found to be significantly negatively associated with overweight and obesity (*p* < 0.05), whereas in girls, only those with not less than moderate activities were negatively associated with overweight and obesity (*p* < 0.05). - Sedentary behaviors, time spent watching television, and time spent working on the computer were not significantly associated with obesity in either sex.
Alqahtani N et al. (14)/KSA	Cross-sectional	370 adolescents (174 females), 14–19 years	PA and ST	Physical-activity questionnaire	Overweight/Obesity	Two-sided tests	- A strong association between moderate to vigorous METs and overweight and obesity among male participants (*p* < 0.001). - Prolonged screen time has a strong association with overweight in males (*p* = 0.01) and females (*p* < 0.001). - Association found between screen time and obesity in males and females (*p* < 0.001).
Al-Hazzaa et al. (24)/KSA	Cross-sectional	2,908 secondary school students (females: 1,507) aged 14–19 years	ST and PA	**ATLS research instrument** - The International Obesity Task Force (IOTF) age- and sex-specific BMI cutoff reference standards - American Academy of Pediatrics guidelines of a maximum of 2 h/d	Dietary habits	- Two-way ANOVA with Bonferroni test - Pearson correlation	−84% of males and 91.2% of females spent more than 2 h on screen time daily and almost half of the males and three-quarters of the females did not meet daily physical activity guidelines. - Screen time was significantly (*p* < 0.05) inversely correlated with the intake of breakfast, vegetables and fruit. Physical activity had a significant (*p* < 0.05) positive relationship with fruit and vegetable intake.
Alghadir et al. (33)/KSA	Cross-sectional	214 adolescents, aged 12 to 18 years	PA and ST	**Self-administered questionnaire that included questions about demographic and anthropometric characteristics, daily after-school routine, physical activity, watching television, using computers, and food preferences**	BMI	Non-parametric (Mann–Whitney U) test	- Saudi boys who reported physical activity 2–5 times per week, the most television time, the most computer time, and the highest frequency of eating fast food and drinking soft drinks had a significantly higher mean body mass index than the non-Saudi boys in their categories
Alzamil et al. (25)/KSA	Cross-sectional	Only female students: *n* = 456,	PA	**The Arab Teen Lifestyle Study (ATLS) questionnaire**	Sleep and dietary habits	- Pearson's correlation - Chi-square test	- Nearly half of the college females were physically inactive. - The majority (>85%) of females spent more time in sedentary activity (>3 h/day) while 95% of females had insufficient sleep (< 8 h/night). - The predictors of total PA time were increased duration of sleep and reduced intake of French fries/potato chips.
Said and Shaab Alibrahim (26)/KSA	Cross-sectional	981 students (Females: 24.46%), 10–15 years	PA and ST	**Bioelectric impedance analysis A questionnaire focusing on lifestyle behaviors over the last seven days**	BMI	Stepwise multiple regression analysis	- Boys were more active, used laptops more frequently, and played more video games than girls. - Girls were less sedentary, watched more TV and used their smartphones more than boys.
Al-Hazzaa and Albawardi (34)/KSA	Cross-sectional	2,888 adolescents: (1,500 females) aged 15–19 years		**The ATLS questionnaire**	Gender and obesity status	ANCOVA and multivariate tests	- Intensive PA showed significant interaction effects between gender and obesity status, whereas the sum of moderate activity energy expenditure, non-leisure-time PA, and sleep duration exhibited significant interaction effects between gender and screen time. - Vegetable intake showed significant three-way interaction effects between gender, waist/height ratio and screen time.
Al-Ghamdi (32)/KSA	Case-control	397 students, 9–14 years	ST	**20-item Arabic questionnaire**	Obesity	Logistic regression analysis	- Higher (body mass index) BMI was associated with a higher number of televisions at home (*P* < 0.001), watching TV for more than 3 h per day at the weekend (*P* = 0.047) - An increase in the number of hours of watching TV over the weekend was significantly associated with an increased risk of childhood obesity.
Kilani et al. (27)/Oman	Cross-sectional	802 adolescents (females: 442), aged 15–18 years	PA	**The ATLS questionnaire**	Eating habits and sleep	- Multinomial logistic regression analysis - Freeman-Tukey's test	- Adolescents had a sedentary lifestyle (lack of PA, average of 6.7 h sleep, and consumption of high-calorie foods). - Males were more than twice as active as females. - 62.5% and 55.5% of males consumed more than three servings of dairy and meat, compared to 18.78 % and 35.2% of females, respectively.
Boulhanna et al. (28)/Morocco	Cross-sectional	812 adolescents: (female: 408) aged 13 to 18 years	PA and ST	**Youth physical activity compendium**	Dietary habits	- Mann–Whitney U-test - Chi-square χ2 or Fisher's test	- The prevalence of physical activity was 36.08% (50.0% among boys and 22.06% among girls). - Nearly 40% of adolescents spend more than 2 h a day in front of the screens. **-** Unhealthy dietary habits were more frequent among adolescents and most of them consume Fried potatoes (fries and chips) Doughnuts/cake, and Sugary drinks more than three times a week.
Al-Thani et al. (9)/Qatar	Cross-sectional	5,862 students (females: 2,924) aged 12 to 17 years	PA and ST	**WHO-Global School Health Survey (GSHS)**	**-**	**-** Spearman's rank correlation, **-** The logistic regression model	**-** Only 35.4% of students were performing 60 min of PA ≥3 days/week. **-** The proportion of students with >2 h screentime ranged from 43% to 57% (weekdays) and 50% to 62.5% (weekends). - Girls had fewer odds of being physically active than boys (odds ratio [OR] = 0.61, *P* < 0.001). - Qatari students were less likely to be physically active than non-Qataris (OR = 0.79, *P* < 0.001).
Kerkadi et al. (29)/Qatar	Cross-sectional	1,161 adolescents (females: 621), 14–18 years	PA and ST	**ATLS questionnaire**	Obesity	**-** Students *t*-test **-** Multivariate logistic regression	**-** Females were more inactive than males (63.7% vs. 36.3%; *p* < 0.001). **-** The proportion of adolescents who reported screen time of over 2 h per day was 82.5%. **-** Females engaged in more sedentary behavior than males (53.4% vs. 46.4%, *p* = 0.009). Being male (OR: 1.3; CI: 1.0–1.7) and skipping breakfast (OR: 1.5; CI: 1.2–2) were significantly associated with overweight/obesity.
Musaiger et al. (30)/Iraq	Cross-sectional	723 adolescents Females: 373, 15–18 years	PA and ST	**ATLS research instrument**	Risk of non-communicable diseases	**-** Independent *t*-tests **-** Chi-square tests	**-** Boys spent more time in physical activity (*p* < 0.001) and looking at screens than girls. **-** There were significant differences between boys and girls in most eating habits and activity behaviors. The frequency of skipping breakfast and the intakes of fruits and vegetables, French fries, and sweets and chocolates were significantly higher among girls than boys (*p* < 0.001).

## 3 Results

The studies included in this review provided results from 59,500 participants from 7 MENA countries: Iran (*n* = 3), Kuwait (*n* = 3), Saudi Arabia (*n* = 7), Oman (*n* = 1), Morocco (*n* = 1), Qatar (*n* = 2), and Iraq (*n* = 1) (Table 1). Of all included studies, 16 were cross-sectional and two were case control. These studies were conducted between 2012 and 2022 and included children and adolescents between 6 and 19 years of age. Sample sizes ranged from 79 to 23,043 participants. Out of the 18 studies, 6 reported data on obesity/overweight, 6 on eating and dietary habits, 2 on BMI, one on quality of life, one on self-rated health, and one on risk of non-communicable diseases. All studies used a reliable and/or valid tool to assess physical activity, sedentary behaviors i-e ST and health outcomes. It was not possible to conduct a meta-analysis due to the heterogeneity of the data, therefore narrative syntheses were conducted.

### 3.1 Prevalence of PA and ST in the MENA region

In a longitudinal cross-sectional study, 40.9% of the students (6–18 years) had excessive screen time (ST greater than 2 h/day) in Iran (21). Moreover, 21.9% of boys reported more than 2 h of screen time compared to 15.2% of girls while 71.2% of boys and 60.4% of girls reported moderate to high physical activity in Iran (12). Another study reported that participants with sufficient activity were 59.09%, and those with insufficient activity were 40.90% while 47.28% of the participants spent >2 h a day on television, video watching and electronic games playing (17). In another study, 44.6% of the boys and 76.0 % of the girls did not meet the recommended daily physical activity levels (≥2,520 MET-min/week, moderate to vigorous intensity). Moreover, 96.3 % of boys and 96.7 % of girls reported spending >2 h/d on screen time in Kuwait (23). A study conducted in KSA showed that 84% of males and 91.2% of females spent more than 2 h of screen time daily and almost half of the males and three-quarters of the females did not meet daily physical activity guidelines (24). Similarly, another study demonstrated that half of the college females were physically inactive. The majority (>85%) of females spent more time in sedentary activity (>3 h/day) (25). Furthermore, boys were more active, used laptops more frequently, and played more video games than girls. However, girls were less sedentary, watched more TV and used their smartphones more than boys (26). A study in Oman revealed that study subjects had a sedentary lifestyle (lack of PA) while males were more than twice as active as females (27). The prevalence of physical activity was 36.08% (50.0% among boys and 22.06% among girls). However, nearly 40% of these adolescents spend more than 2 h a day in front of screens (28). Furthermore, a study conducted in Qatar, demonstrated that 35.4% of students were performing 60 min of PA ≥3 days/week. The proportion of students with >2 h screentime ranged from 43% to 57% (weekdays) and 50% to 62.5% (weekends). Girls were less physically active than the boys while Qatari students were less likely to be physically active than non-Qataris (9). In another study, female adolescents were more inactive than males (63.7% vs. 36.3%; *p* < 0.001). The proportion of adolescents who reported screen time of over 2 h per day was 82.5%. However, females engaged in more sedentary behaviors than males (53.4% vs. 46.4%, *p* = 0.009) (29). Similarly, a study in Iraq reported that boys spent more time in both physical activity (*p* < 0.001) and screen use than girls (30). Overall, the proportion of >2 h/day screen time ranged from 21.9% to 96.7%, indicating high sedentary behaviors and low PA across MENA. Boys were generally more active and had less screen time than girls.

### 3.2 Association of PA and ST with eating habits and health outcomes

It has been shown that ST duration had a significant inverse association with total QoL (β: – 0.49, *p* < 0.05) while PA showed positive significant associations with HRQoL total score (β: 1.8, *p* < 0.05) among students in Iran (21). More physical activity was associated with good self-rated health (OR 1.37) and life satisfaction (OR 1.39), while prolonged screen time was not associated with good self-rated health (OR 1.02) and life satisfaction (OR 0.94) (12). Children (10–12 years) who spend more time on ST activities, independent of their physical activities, are more susceptible to overweight and obesity in Iran (*p* = 0.002) (17). In a study on adolescents (14–19 years) in Kuwait, among boys, moderate and vigorous activities were found to be significantly negatively associated with overweight and obesity (*p* < 0.05), whereas in girls, only those with not less than moderate activities were negatively associated with overweight and obesity (*p* < 0.05). Sedentary behaviors including time spent watching television and working on the computer was not significantly associated with obesity in either sex (31). A strong association was found between moderate to vigorous METs and overweight and obesity among male participants (*p* < 0.001). In addition, prolonged screen time has a strong association with overweight and obesity in male (*p* = 0.01) and female (*p* < 0.001) adolescents in KSA (32). Screen time was significantly (*p* < 0.05) inversely correlated with the intake of breakfast, vegetables, and fruit while physical activity had a significant (*p* < 0.05) positive relationship with fruit and vegetable intake (24). Saudi boys who reported physical activity 2–5 times per week, the most television time, the most computer time, and the highest frequency of eating fast food and drinking soft drinks had a significantly higher mean body mass index than the non-Saudi boys in their categories (33). The predictors of total PA time were increased the duration of sleep and reduced the intake of French fries/potato chips (25). Intensive PA showed significant interaction effects between gender and obesity status, whereas the sum of moderate activity energy expenditure, non-leisure-time PA, and sleep duration exhibited significant interaction effects between gender and screen time. Vegetable intake showed significant three-way interaction effects between gender, waist/height ratio and screen time (34). Higher BMI was associated with a higher number of televisions at home (*p* < 0.001), and watching TV for more than 3 h per day at the weekend (*p* = 0.047). An increase in the number of hours of watching TV over the weekend was significantly associated with an increased risk of childhood obesity (32).

## 4 Discussion

Our review incorporated findings from 18 studies conducted in 7 different MENA countries. It was observed that a high percentage (on average more than 50%) of children and adolescents in the MENA region didn't meet the recommended daily physical activity (>60 min/day). The most frequently cited obstacles to getting regular exercise are lack of time, social support and motivation, gender and cultural norms, and hot weather. Reduced levels of physical fitness, mental health issues, and delays in social development have all been linked to excessive time spent in front of screens. Exposure to screens for long periods may increase the risk of childhood overweight/obesity due to lack of physical activity (35). Moreover, the number of studies that looked at obesity risk factors in MENA nations was also minimal, but a systematic review published in 2017 revealed that higher social status, increased screen time, and physical inactivity were all risk factors for childhood obesity. The prevalence of childhood and adolescent obesity is a significant problem for many nations in the MENA region (36).

In Saudi Arabia, a study showed that teenagers in Saudi Arabia spent a significant amount of time in front of screens (84% of boys and 91.2% of girls) each day, and about 50% of males and 75% of females met the recommended amounts of physical activity each day. Females in Saudi Arabia (Al-Khober, Jeddah, and Riyadh) were substantially more sedentary and less active than males (*p* = 0.001) (24). Higher physical activity was found to be significantly associated with higher consumption of fruits, vegetables, milk, and energy drinks in logistic regression analyses, and higher screen time (ST) was found to be significantly associated with higher consumption of sugar-sweetened drinks, fast foods, cake/doughnuts, and energy drinks (37). Another study supports such finding conducted in Iraq, that girls skipped breakfast more frequently than boys, and their intakes of fruits and vegetables, french fries, and sweets and chocolates were significantly greater (*p* < 0.001). Boys were much more likely than girls to consume fast foods, sugar-sweetened beverages, and energy drinks but spent more time engaging in physical activity (*p* < 0.001) and viewing screens than girls (30).

Another investigation demonstrated the relationship between television viewing and childhood obesity in Saudi children aged 9 to 14 and discovered that having only one television at home was related to a 42% reduction in the risk of childhood obesity (OR = 0.58, *p* = 0.001). Furthermore, personal ownership of television by a child was associated with an increased risk of obesity (OR = 1.75, *p* = 0.002). This study also discovered that reducing the number of hours spent viewing television on weekends by 1 h resulted in a 19% reduction in the risk of obesity (OR = 0.81, *p* = 0.009). In contrast, Al-Ghamdi investigated that personal computers and the Internet are not significantly associated with an increased risk of childhood obesity (32). Another study among rural adolescents found that prolonged screen time is associated with overweight in both men (*p* = 0.01) and women (*p* < 0.001). Screen time and obesity were shown to have a similar relationship in both men and women (*p* < 0.001) (14). The main sedentary activities that influenced BMI were using laptops and playing video games (26). A cross-sectional study conducted among females attending health science colleges showed that almost half of female students were physically inactive. Females exercised primarily at home or alone at no specific time of day. Their activity was motivated by health (43.4%) and weight loss (28.7%), with lack of time (71.2%) being the leading reason for inactivity (25).

Although the MENA region is grappling with issues related to physical activity and obesity, which stem from many lifestyle variables, such patterns are visible in other nations as well, creating serious public health implications. Over the previous 40 years, the frequency of pediatric obesity in the US has more than tripled, rising from 5% in 1978 to 18.5% in 2016 (38). Preschoolers had a lower prevalence of obesity (13.9%) compared to teenagers (12–19 years; 20.6%) and school-aged children (6–11 years; 18.4%). Obesity was more common among boys in school (20.4%) than among boys in preschool (14.3%). The prevalence of obesity was greater among teenage females (20.9%) compared to preschool-aged girls (13.5%) (39).

The establishment of healthy lifestyle practices is essential during adolescence and young adulthood to maintain activity levels and prevent obesity (40). From 1998 to 2009, researchers in the US, Europe, Australia, and Brazil found that boys engaged in moderate-to-vigorous-intensity physical activity (MVPA) for approximately 55% of their waking hours, a substantial advantage over girls (41). In the United Kingdom, a study indicated that moderate to vigorous physical activity (MVPA) and extended sleep duration correlated with reduced odds of overweight or obesity, while sedentary time (ST) and a healthy diet score were linked to heightened probabilities of overweight or obesity (42). Another study conducted in the United States, indicates that positive wellbeing in adolescence reflects improved reported overall health and a reduction in dangerous health behaviors in young adulthood. Following the objectives of the positive youth development framework, fostering, and cultivating positive wellbeing during the transition from childhood to adolescence may offer a viable approach to enhancing long-term health (43).

Sedentism and a lack of physical activity, as well as high consumption of high-fat fast foods and sugary drinks, endanger the health of children and adolescents in the MENA region & other countries. To encourage kids to lead healthy lives, it is important to use tech-driven solutions like gamified exercise programs and interactive health apps. Furthermore, healthcare providers and educational institutions should work together to design intervention programs that stress the need for regular exercise, healthy eating, and appropriate use of technology. Society may promote a better environment for kids and teens by adopting these measures, which will have positive effects on public health and people's quality of life in the long run. Additional research needs to be done to record nationally comparable prevalence rates, track the problem's trends, understand the environmental risk factors in each country adapt a strategy according to these risk factors, and test appropriate interventions for their efficacy in the management of the issue.

A significant weakness of this review is the absence of discourse on the diversity of measurement instruments employed in the included studies to evaluate physical activity and sedentary behavior. The lack of standardized, objective instruments such as accelerometers restricts the comparability of results. Numerous researches depend on self-reported questionnaires, which are susceptible to recall bias and exaggeration. Research indicates that the lengthy form of the International Physical Activity Questionnaire (IPAQ) considerably overestimates physical activity levels in comparison to its short form and, crucially, to objective measures such as the ActiGraph. Methodological discrepancies impede the capacity to derive reliable and generalizable results. Another limitation of this review is that it incorporated data on the prevalence of physical activity and screen time from MENA studies, although aggregation was not feasible. The studies varied significantly in several aspects, including definitions and thresholds for physical activity and screen time, measurement instruments (e.g., self-reported questionnaires vs. device-based evaluations), age group categorizations, and reporting formats. These variations hinder direct comparability and the appropriate summary of prevalence estimates.

## 5 Conclusion

This review demonstrates that most children and adolescents are involved in less physical activity and spend more time on screening and sedentary behaviors. Moreover, girls are less active than boys. In the MENA region, childhood sedentary behaviors like physical inactivity and more screen time are significant risk factors for developing NCDs including obesity, diabetes, cardiovascular disease, and cancer in later life. Given the inadequate levels of physical activity in the Arabian Peninsula and high levels of sedentary behavior, a substantially better evidence-based intervention program is required to improve the quality of life of children and adolescents and reduce the burden of NCDs at the national level. PA interventions that are country-, sociocultural-, and environmental-specific are required. Given the high rate of obesity among adolescents in the Arab world, as well as an environment that promotes an unhealthy lifestyle and eating culture, it is critical to investigate the barriers to healthy living.

## Data Availability

The original contributions presented in the study are included in the article/supplementary material, further inquiries can be directed to the corresponding author.
